# Fostering Empathy in Patient-Medical Student Relationships During a 48-Hour Hospice Home Immersion

**DOI:** 10.1093/geroni/igaf122.4123

**Published:** 2025-12-31

**Authors:** Brandon Asika Etuka, Sophia Moschella, Jhanvi Puri, Marilyn Gugliucci

**Affiliations:** University of New England College of Osteopathic Medicine, Middletown, Connecticut, United States; University of New England, Biddeford, Maine, United States; University of New England College of Osteopathic Medicine, Portland, Maine, United States; University of New England College of Osteopathic Medicine, Kennebunk, Maine, United States

## Abstract

While studies show that empathy declines throughout a medical student’s clinical education, fewer than one-third of medical schools have dedicated course content focusing on providing compassionate end-of-life care. Our analysis through the University of New England College of Osteopathic Medicine’s “Learning-by-Living: 48-Hour Hospice Home Immersion Project” strived to investigate how second-year medical students’ empathy is impacted during this unique hospice patient care immersion. Phenomenologic qualitative analysis was applied to identify themes from accounts expressed in ten student immersion journals written during AY 2020-2021. Manual content data analysis was conducted for each journal, which was the foundation for creating a codebook from which inter-rater reliability was based. Thematic coding of the journal data was conducted manually and then fine-tuned using NVivo-15 software to categorize quotes within each theme. Through analyses, 43 themes were identified with significant quotes stored within each. The top four themes addressing medical students’ empathy were: Impact of a Patient’s Story on Empathy, Preservation of Patients’ Dignity and Humanity, Building an Emotional Toolbox, and Empathy and Appreciation for Colleagues and Caregivers. Students learned from the personal stories and experiences of patients and their families along with working with the interprofessional hospice team. Student reflections demonstrated significant understanding of the services hospice provides. They also gained self-awareness and identified skills applicable to their careers as future physicians, including navigating open discussions surrounding end-of-life care with patients/families. This project creates impactful learning, skill development, and end-of-life experiences that augment students’ ability to be empathetic providers.

